# The Course of AαVal541 as a Proteinase 3 Specific Neo-Epitope after Alpha-1-Antitrypsin Augmentation in Severe Deficient Patients

**DOI:** 10.3390/ijms22158031

**Published:** 2021-07-27

**Authors:** Iris G. M. Schouten, Richard A. Mumford, Dirk Jan A. R. Moes, Pieter S. Hiemstra, Jan Stolk

**Affiliations:** 1 Department of Pulmonology, Leiden University Medical Center, Albinusdreef 2, 2333 ZA Leiden, The Netherlands; ra.mumford@gmail.com (R.A.M.); p.s.hiemstra@lumc.nl (P.S.H.); J.Stolk@lumc.nl (J.S.); 2 Department of Clinical Pharmacy and Toxicology, Leiden University Medical Center, Albinusdreef 2, 2333 ZA Leiden, The Netherlands; d.j.a.r.moes@lumc.nl

**Keywords:** alpha-1-antitrypsin deficiency, proteinase 3, alpha-1-antitrypsin augmentation therapy, biomarker

## Abstract

In alpha-1-antitrypsin deficiency (AATD), neutrophil serine proteases such as elastase and proteinase 3 (PR3) are insufficiently inhibited. A previous study in AATD patients showed a higher plasma level of the specific PR3-generated fibrinogen-derived peptide AαVal541, compared with healthy controls. Here, we analyzed the course of AαVal541 plasma levels during 4 weeks after a single iv dose of 240 mg/kg AAT in ten patients with genotype Z/Rare or Rare/Rare. To this end, we developed an immunoassay to measure AαVal541 in plasma and applied population pharmacokinetic modeling for AAT. The median AαVal541 plasma level before treatment was 140.2 nM (IQR 51.5–234.8 nM)). In five patients who received AAT for the first time, AαVal541 levels decreased to 20.6 nM (IQR 5.8–88.9 nM), and in five patients who already had received multiple infusions before, it decreased to 26.2 nM (IQR 22.31–35.0 nM). In 9 of 10 patients, AαVal541 levels were reduced to the median level of healthy controls (21.4 nM; IQR 16.7–30.1 nM). At 7–14 days after treatment, AαVal541 levels started to increase again in all patients. Our results show that fibrinopeptide AαVal541 may serve as a biochemical footprint to assess the efficacy of in vivo inhibition of PR3 activity in patients receiving intravenous AAT augmentation therapy.

## 1. Introduction

Alpha-1-antitrypsin (AAT) deficiency is an inherited disorder caused by mutations in the *SERPINA1* gene located on chromosome 14. Various mutations have been identified that cause reduced serum levels of AAT, and the most common mutations are the missense mutations (Glu342Lys) Z and (Glu264Val) S. These mutations result in serum AAT concentrations of 15% in patients with homozygous ZZ mutations and 33.3% of normal levels in heterozygous SZ mutations, respectively [1]. Very low circulating AAT levels can be caused by mutants with a stop codon in *SERPINA1* (null alleles, like Q_0Bellingham_) or by mutants coding for synthesis of a misfolded protein that is rapidly degraded by the endoplasmic reticulum (ER)-associated machinery (M-like alleles, such as M_Procida_ [2] and M_Heerlen_ [3]). Previously, we showed that serum levels of AAT are correlated with the severity of emphysema [4]. Subjects with null mutations should be considered a subgroup at particularly high risk for developing emphysema. Conventionally, this is linked to the protease–antiprotease hypothesis, which indicates that a deficiency of AAT, the most important antiprotease in the lung, results in impaired inhibition of its targets, neutrophil elastase (NE) and proteinase 3 (PR3) in lung tissue [5]. This unrestricted activity leads to destruction of alveoli, the hallmark of emphysema in AAT deficiency. Although previous research about the pathophysiology of emphysema in AATD has mainly focused on NE activity in AATD, PR3 is also considered as an important neutrophil serine protease involved in the pathophysiology of emphysema [6,7].

Intravenous AAT augmentation therapy with a dose of 60 mg/kg was introduced based on the hypothesis that restoring the protease–antiprotease balance through AAT augmentation causes a reduction of pulmonary function decline expressed as forced expiration volume in one second (FEV_1_) [8]. However, only a limited number of studies of airways secretions indicate that a rise in local AAT levels and associated reduction in protease activity supports this concept [9]. Importantly, it has been difficult to prove the efficacy of AAT therapy in randomized clinical trials with FEV_1_ as outcome parameter.

More recent studies suggested that newly developed biomarkers or footprints of excess protease activity might be useful when performing dose-ranging studies of AAT augmentation therapy to evaluate its effect on the protease balance and to determine whether the current dose of 60 mg/kg is sufficient for an individual patient, as proposed by Campos et al. [10]. Newby et al. described a footprint of PR3 activity, the fibrinopeptide AαVal541, that is a neoepitope generated by PR3-mediated cleavage of fibrinogen [11]. They showed a reduction of plasma AαVal541 levels in AATD patients after six months of augmentation therapy to levels slightly higher than the AαVal541 they found in healthy controls. Furthermore, they found that the circulating levels of the AαVal541 epitope were stable over time in untreated PiZZ patients. This shows that the AαVal541 epitope is sensitive to AAT augmentation therapy, but the variability after augmentation therapy between two doses is still unknown.

In this study, we aimed to investigate the time course of levels of the fibrinopeptide AαVal541 over a month following a single dose of AAT augmentation therapy in severe deficient AATD patients by pharmacokinetic modeling to evaluate the inhibitory effect of AAT on PR3.

## 2. Results

### 2.1. AαVal541 Assay Development and Validation

Antisera against AαVal541 was generated in two rabbits. Rabbit 972 was selected for further use in the assay (Supplementary Material and Figure S1).

Several experiments were performed to demonstrate the specificity of the antiserum for the AαVal541 epitope. First, we showed that there was no binding of the antibody to uncleaved fibrinogen and only little binding to NE-cleaved fibrinogen (Supplementary Material and Figure S2). Second, we found the valine residue at the N-terminal of the AαVal541 peptide to be crucial for detection of the AαVal541 antibody (Figure S3).

**Figure 1:**
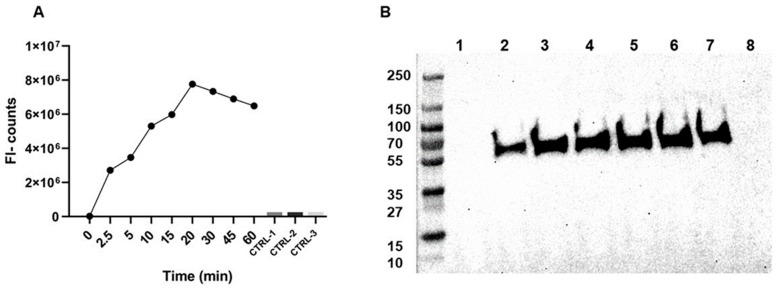
Time-dependent formation of the AαVal541 epitope by PR3 cleavage of fibrinogen. The AαVal541 epitope in fibrinogen was generated by incubating fibrinogen with PR3 for different periods of time. The cleavage reaction was stopped by adding AAT. (**A**) Samples were coated to a 96-well plate and incubated with the AαVal541 antibody, and binding is indicated by fluorescence (see Materials and Methods for details). Three control samples were included. CTRL-1: preincubation of PR3 with AAT for 30 min before adding fibrinogen. This sample was directly placed on ice after adding fibrinogen. CTRL-2: preincubation of PR3 with AAT for 30 min before the incubation with fibrinogen for one hour. CRLT-3: incubation of fibrinogen alone for one hour without PR3 or AAT. (**B**) Detection of generation of AαVal541 by Western blot analysis of the different samples of PR3-cleaved fibrinogen obtained at different time points using the AαVal541 antibody. Molecular weight of protein ladder is shown on the left. Lane 1: control, only fibrinogen; Lanes 2–7: incubation of PR3 with fibrinogen for 2 min (lane 2), 5 min (lane 3), 10/15 min (lane 4), 20 min (lane 5), 45 min (lane 6) or 60 min (lane 7); lane 8: control using PR3 preincubated with AAT before addition to fibrinogen and incubation for 60 min. Abbreviations: FL-counts = fluorescence counts.

For the development of the AαVal541 immunoassay, first the cleavage reaction of fibrinogen and generation of the AαVal541 neo-epitope by PR3 was evaluated. The generation of the AαVal541 neo-epitope was time-dependent (Figure 1). The samples of timepoints 10 and 15 min were pooled for further assay development.

**Figure 2:**
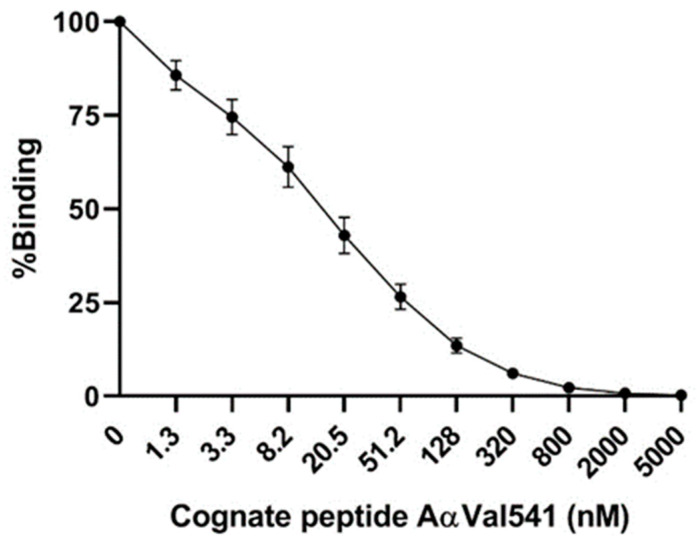
Day-to-day variability of the AαVal541 immunoassay. Twenty different standard curves were generated on 20 different days. The AαVal541 cognate peptide was pre-incubated with the AαVal541 antibody before addition to the PR3-cleaved fibrinogen-coated plate. The mean (±SD) of 20 standard curves of the immunoassay are shown. The average calculated value of the 20 curves showed an EC20 value of 87.7 nM (95% CI 79.8–96.8 nM), EC50 of 14.6 nM (13.7–15.6 nM) and EC80 of 2.4 nM (2.2–2.7 nM).

In a next series of experiments, we demonstrated the developed AαVal541 immunoassay to have limited variability between days (Figure 2).

### 2.2. Study Population and Baseline Characteristics

For this study, 10 AATD patients were analyzed. Of these 10 patients, 5 already received monthly AAT infusions multiple times before, and 5 patients had not received AAT infusion previously. The characteristics of the patients are summarized in Table 1.

### 2.3. AαVal541 Levels before Start AAT Augmentation Therapy

**Figure 3:**
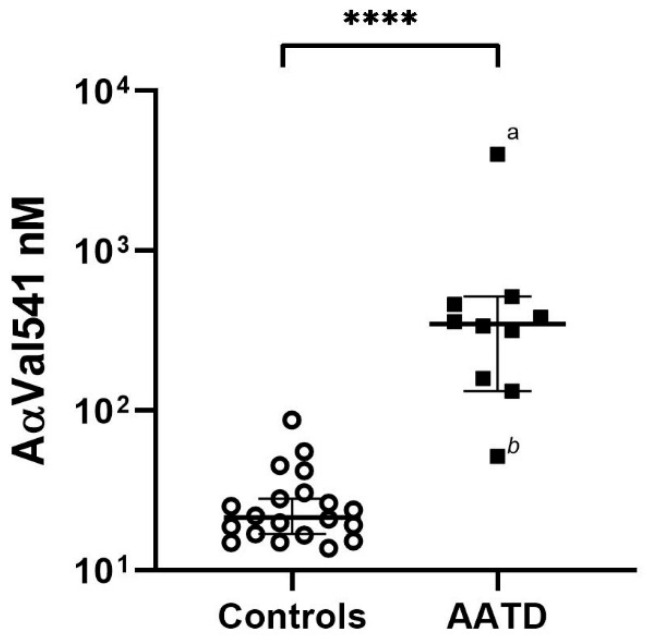
Higher levels of AαVal541 in AATD patients compared with healthy controls. The median (IQR) AαVal541 level in the AATD patients at the moment of assessment for eligibility for starting AAT augmentation therapy (*n* = 10) was 348.3 nM (152.4–475.8 nM) and 21.4 nM (16.7–30.1 nM) in healthy controls (*n* = 20). ^a^ represents an AATD patient with an FEV_1_ of 17%predicted and KCO of 18%pred. ^b^ represents an AATD patient with an FEV_1_ of 57%pred and KCO of 75%pred. ****, significant difference *p* < 0.0001, Mann–Whitney U test.

First, from all patients, ‘baseline’ AαVal541 level was measured at the assessment for eligibility for starting AAT augmentation therapy, and values were compared with those of healthy controls. Figure 3 shows that the AαVal541 levels in the 10 AATD patients before starting AAT augmentation therapy were significantly higher compared with the age-matched non-smoking healthy control group (*n* = 20).

The pre-administration baseline AαVal541 level of the AATD patients before they received their first AAT augmentation therapy showed no correlation with baseline pulmonary function, although diffusion capacity (KCO) showed a trend to a negative correlation with baseline AαVal541 level (Spearman’s correlation coefficient—0.624, *p* = 0.054).

### 2.4. The Effect of AAT Infusion on AαVal541 Levels

**Figure 4:**
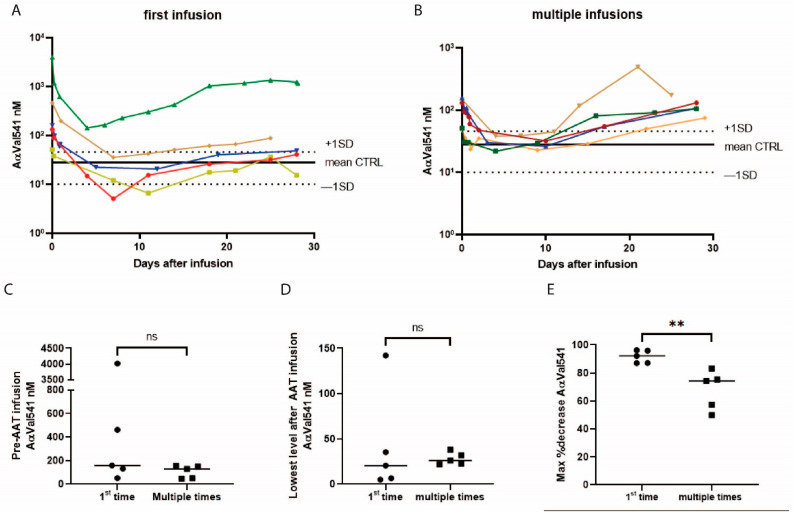
The AαVal541 levels decrease to the average range of healthy controls after a single dose of AAT. (**A**) The course of the AαVal541 plasma level in 5 patients who received AAT augmentation therapy for the first time are shown, as well the average AαVal541 of the 20 healthy controls (black horizontal line). (**B**) The course of the AαVal541 level in 5 patients who already received AAT augmentation therapy in the months before. (**C**) The AαVal541 plasma levels before the single dose of 240 mg/kg AAT of patients who received AAT infusion for the first time and patients who received AAT infusion before. (**D**) The lowest AαVal541 value after 240 mg/kg AAT in patients who received AAT infusion for the first time and patients who received AAT infusion before. (**E**) The maximum percentage of AαVal541 decrease with respect to the AαVal541 level prior to the monthly dose of AAT infusion both groups. ns, non-significant difference, Mann–Whitney U test, **; significant difference *p* = 0.0079, Mann–Whitney U test.

To study the effect of AAT augmentation administration, the AαVal541 levels after infusion were analyzed. The median AαVal541 plasma level before the single dose AAT infusion in the five patients who received AAT infusion for the first time and the five patients who already received AAT infusions multiple times before was in the range of 140.2 nM (IQR 51.5–234.8 nM). In all patients, the AαVal541 level decreased within one day after iv AAT, and over time, the levels were further reduced to the median level in healthy controls of 21.4 nM (IQR 16.7–30.1 nM) in 9 of 10 patients. At approximately 7–14 days after treatment, AαVal541 levels started to increase again in all patients, reaching a median of 96.4 nM (IQR 46.6–141.4.0 nM) at about 4 weeks after the AAT dose (Figure 4A,B and Figure S4). In the five patients who received AAT for the first time, the AαVal541 decreased to 20.6 nM (IQR 5.8–88.9 nM) of the baseline AαVal541 value. In the patients (*n* = 5) who already received AAT infusions before, the AαVal541 decreased to 26.2 nM (IQR 22.31–35.0 nM). However, in all patients who had received multiple infusions, AαVal541 levels above the healthy control and some even higher than before the AAT infusion were detected in the last sample collected before the next infusion, while this was only seen in two out of five patients receiving AAT infusion for the first time. There was no significant difference in the AαVal541 level before the AAT infusion between the two groups and no significant difference in absolute AαVal541 level decrease but the AαVal541 decrease in percentage of baseline decreased significantly stronger in the patients who received AAT infusion for the first time than in those who had received multiple treatments before (Figure 4C–E).

### 2.5. Pharmacokinetic Modelling AAT after Single Infusion

To analyze the effect of a single dose of 240 mg/kg AAT on the AαVal541 level, population pharmacokinetic modelling was applied. The pharmacokinetic parameters of AAT were estimated from a two-compartment model in which a proportional error was applied (Table 2). The average T_1/2_ of AAT was 7.3 days (range 5.0–10.4 days). In Table 3, the pharmacokinetic parameters of each individual patient are summarized.

### 2.6. Pharmacokinetic Modelling of AAT and AαVal541 Levels

**Figure 5:**
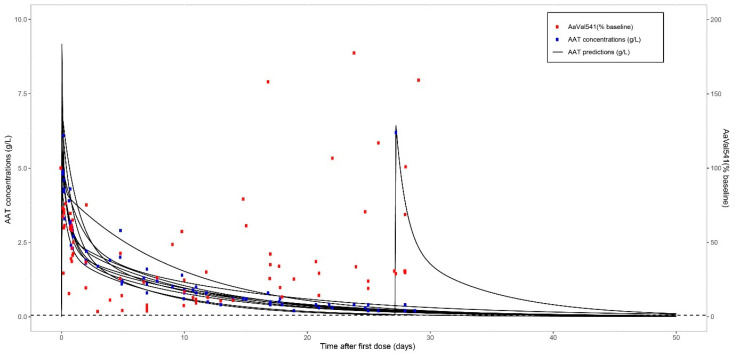
Summary of the course of the AAT and AαVal541 levels after one monthly dose of 240 mg/kg AAT in 10 AATD patients. Left *y* axis plots the AAT levels with both the predicted AAT levels by applying two-compartment pharmacokinetic model (black line), and the measured (blue dots) AAT levels at different time points over a month; the right *y* axis plots the AαVal541 levels (red dots) expressed as percentage of baseline (AαVal541 before the infusion) measured on the different time points. One AATD patient received another AAT infusion at day 28.

Figure 5 shows both the measured and model-predicted levels of AAT of each patient plotted together with the AαVal541 levels in percentage of individual baseline levels. Directly after the administration of AAT, the AαVal541 levels started to decrease. However, after the AAT level reached levels below approximately 1.25 g/L, an increase in AαVal541 levels was observed (Figure 5 and Figure S5).

**Figure 6:**
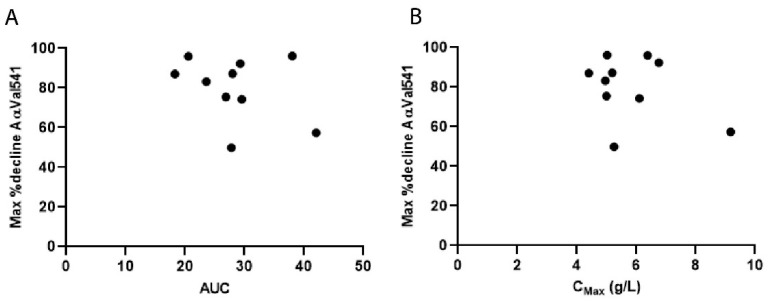
Correlation between AUC_0-inf_ and maximum %decrease of AαVal541 and correlation between C_max_ and maximum %decrease AαVal541. In (**A**), the maximum decrease of AαVal541 after administration of the monthly dose of AAT in percentage of the AαVal541 level prior to the infusion is plotted against the C_max_. No correlation was found between C_max_ and maximum %decrease of AαVal541. (Spearman coefficient = −0.103, *p* = 0.777). In (**B**), the maximum decrease of AαVal541 after administration of the monthly dose of AAT in percentage of the AαVal541 level prior to the infusion is plotted against AUC_0-inf_. Each dot represents an individual patient. There was no significant correlation of AUC_0-inf_ with the maximum %decrease of AαVal541 (Spearman coefficient = −0.1273, *p* = 0.7330). Abbreviations: AUC_0-inf_ = area under the AAT plasma concentration-time curve from dose administration to infinity, C_max_ = Peak plasma concentration of AAT in g/L.

There was no correlation between the area under the curve (AUC_0-inf_) and the maximal decrease of AαVal541 and no correlation between the maximum decrease of AαVal541 and C_max_ (Figure 6). There was also no correlation found between the AUC_0-inf_ and weight or AUC_0-inf_ and age (Figure S6).

## 3. Discussion

For the current study, we successfully developed an immunoassay to measure the PR3-specific fibrinopeptide AαVal541 in plasma and demonstrated how it can be used as a biomarker of PR3 activity. Using this immunoassay that detects the footprint of PR3 activity, we provide evidence that in AATD patients a single dose of 240 mg/kg AAT results in a marked transient reduction in PR3 activity, reaching levels of non-smoking AAT sufficient controls. Following this decrease of AαVal541 after AAT administration, after approximately 7–14 days, levels increase again at a time when AAT levels decrease. Population pharmacokinetic modelling provided novel insight into the value of this new biomarker for PR3 activity and its potential use in monitoring the effects of AAT augmentation therapy.

Here, we used an assay to detect AαVal541 levels as a marker of PR3 activity based on principles described in previous studies [11]. We applied this assay to monitor levels of AαVal541 in between two doses of AAT, which has not been previously reported. Others showed that the PR3- and NE-specific fibrinopeptides were reduced after six months of weekly 60 mg/kg AAT augmentation [11,12]. Campos et al. reported that the NE-specific fibrinopeptide AαVal360 showed a significantly stronger decrease in AATD patients who received 120 mg/kg weekly AAT infusion compared with those who received 60 mg/kg weekly AAT infusion [10]. However, it has not been reported that the AαVal541 levels already decrease shortly after a single infusion and decrease to the levels of non-smoking healthy controls in between doses. Moreover, we applied the combination of population-based pharmacokinetics for 240 mg/kg AAT augmentation and our biomarker of PR3 activity. The concentration-time data was best described using a two-compartment model with linear elimination, which is in line with previous publications [13,14]. Using this model, we found a half-life estimate of AAT of 7.2 days (range 5.0–10.4 days). Additionally, we showed that by modeling the pharmacokinetics of AAT, the AαVal541 level started to increase again when AAT levels are decreased by approximately 50%.

Our study has some important strengths. First, we were able to develop a reproducible and specific immunoassay. Reproducibility was demonstrated by the low standard deviation of twenty standard curves of the immunoassay. Use of the polyclonal antibody that was generated for this immunoassay resulted in a sensitive immunoassay with a lower limit of detection of 2.4 nM. We considered generating a monoclonal antibody but decided to focus on the polyclonal antibody since other investigators appeared to have failed to generate such a monoclonal antibody for detection of AαVal541 (personal communication RM). Second, our study patients had extremely low AAT levels in their circulation due to presence of rare mutations in the *SERPINA1* gene, and this allowed accurate monitoring of the effect of AAT administration on our PR3 biomarker by rendering them vulnerable to producing high levels of AαVal541. Third, by applying population-based pharmacokinetic modelling, it was possible to generate estimated pharmacokinetic parameters in a small group of patients.

However, there are also some limitations to our study. Before the development of the AαVal541 immunoassay was finished, some patients had already started on treatment with 240 mg/kg AAT; hence, there was variability in the number of monthly AAT infusions prior to the blood sampling. Another limitation is that the use of AαVal541 in pharmacodynamic modelling, in contrast to the population-based pharmacokinetic modelling, to generate reliable parameters was not possible due to the low number of included patients. Furthermore, despite the apparent specificity of the AαVal541 antibody, the analysis by Western blot and the immunoassay showed that NE-mediated cleavage of fibrinogen also generates low levels of the AαVal541 epitope in fibrinogen. Nevertheless, because the amount of AαVal541 generated by NE is rather small, it was not considered to contribute to measurement of the AαVal541 levels in plasma. Furthermore, we assume that at the moment of neutrophil activation, i.e., PR3 release, the fibrinopeptide AαVal541 is generated and thus assume that AαVal541 plasma levels reflect the inhibitory effect of AAT on PR3. Since both NE and PR3 contribute to neutrophil-mediated lung injury, we did consider studying the effect of AAT on the NE fibrinopeptide AαVal360 as well. However, several polyclonal antibodies raised by our vendor to detect fibrinopeptide AαVal360 in a similar immunoassay were unfortunately not suitable to develop sufficiently sensitive assays for this NE activity biomarker.

To interpret plasma AαVal541 values and their modulation following iv AAT treatment in our patients, detectable AαVal541 plasma levels in healthy non-smoking controls need to be taken into account. First, it is possible that part of this AαVal541 was generated during blood collection and plasma preparation due to inadvertent neutrophil activation, despite careful blood collection and quick processing of the samples to avoid neutrophil stimulation. However, this cannot explain why the AαVal541 level in AATD patients after 240 mg/kg did not decrease below the –1SD level of the average of healthy controls. Secondly, we cannot formally exclude the possibility that proteases other than PR3 contributed to the generation of the AαVal541 epitope. Third, AαVal541 might be generated in part in the near vicinity of the degranulating neutrophil, where active protease levels have been suggested to exceed the levels of inhibitors such as AAT [15]. Campbell et al. later showed that this may in part be explained by the fact that membrane-bound PR3 is not inhibited by AAT [16], suggesting that membrane-bound PR3 may still generate AαVal541 by cleaving fibrinogen. Finally, neutrophil activation and accompanying release of proteases such as NE and PR3 also occur in AAT-sufficient subjects, as shown by the presence of AAT-NE and AAT-PR3 complexes in their serum [12,17].

In our study, we measured a significant difference in percentage decrease of AαVal541 relative to pre-infusion AαVal541 values between patients who received AAT augmentation therapy for the first time and those who already received AAT augmentation up to 5 times, although the lowest level of AαVal541 after infusion did not differ between those patients and fell in the range of healthy controls. This may imply that directly after the first dose of AAT infusion, all PR3 activity is completely inhibited. To further study this effect, pharmacodynamic modelling of AAT with AαVal541 is needed. Because of the low number of included patients, pharmacodynamic modeling of AAT with AαVal541 unfortunately could not reliably be performed in this study. The pharmacokinetic modelling of 240 mg/kg dose AAT showed that between 7–14 days after AAT infusion, the AαVal541 levels started to increase again, suggesting a decrease in inhibition of PR3. Whether this is related to the known higher affinity of AAT for NE compared with PR3, resulting in differential inhibition of these two proteases when AAT levels become limiting, requires further investigation. Additionally, in some of the patients, we even observed an increase in AαVal541 plasma level to levels above the pre-infusion level. This could be explained by a variation in PR3 activity over time. Newby et al. showed some, although not significant, long-term variation (baseline median 281.4 nM (interquartile range (IQR) 168.4–332.9 nM) and after 12–24 months median 215.2 nM (IQR 135.6–352.9 nM)) in 39 PiZZ AATD patients who were naïve for AAT iv treatment [11]. Additionally, it is possible that variable AαVal541 levels reflect the number of circulating neutrophils.

The AαVal541 levels of the 10 included AATD patients in plasma obtained before they ever received AAT augmentation therapy did not significantly correlate with pulmonary function. There was a trend towards a negative correlation with KCO %predicted, which was not significant but which may be related to the low number of subjects included. Indeed, Newby et al. found a significant, though much lower negative correlation of baseline AαVal541 level and KCO %predicted in a larger population (*n* = 233) of PiZZ patients [11].

The current dose of weekly 60 mg/kg AAT augmentation therapy recommended in guidelines has not been demonstrated to preserve lung function in AATD patients. However, a small effect on lung densitometry has been reported [18,19,20]. Therefore, it is important to evaluate the effect of the historically introduced weekly dose of 60 mg/kg on other readouts than solely pulmonary function. Our present findings and those of Newby et al. [11], showing the use of AαVal541 as a biomarker of PR3 activity, may contribute to defining AAT doses that keep AαVal541 values within the range measured in plasma of healthy controls, and thus may prevent further reduction in function in AATD patients.

## 4. Materials and Methods

### 4.1. Study Design and Subjects

In this cohort study, AαVal541 levels were monitored following a monthly dose (240 mg/kg) of AAT augmentation therapy. For this study, plasma samples and lung function data of 10 AATD patients were collected. Pulmonary function testing was performed, and blood samples were collected as part of regular clinical care. The protocol was approved by the local Medical Ethics Committee. Patient characteristics are summarized in Table 1.

In 2017, the Dutch health care providers approved reimbursement of AAT augmentation therapy (Respreeza^®^, CSL Behring, Marburg, Germany) for Z/Null and Null/Null deficient patients, under the condition of close monitoring and yearly evaluation by pulmonary function testing. Some supply issues for Respreeza^®^ occurred since the introduction of AAT augmentation therapy in the Netherlands. For this reason, some AATD patients temporary received Prolastin^®^ (Grifols, Barcelona, Spain) instead. During the outpatient clinic visit for assessment for eligibility for starting AAT augmentation therapy, patients underwent pulmonary function assessment, which included post-bronchodilator spirometry, diffusion capacity of the lungs for carbon monoxide, six-minute walk test and blood sampling. All pulmonary function assessments were performed following the ERS/ATS standards [21,22,23] at the outpatient clinic. All patients started with monthly AAT infusions (240 mg/kg), and after several monthly infusions they were switched to weekly (60 mg/kg) or biweekly (120 mg/kg) infusions. Several plasma samples were collected during one month after a monthly infusion of AAT augmentation therapy to measure AAT and AαVal541 levels.

For the determination of the ‘healthy’ average level of the AαVal541, samples from healthy non-smokers with an age above 40 years were included. These samples were provided by the Leiden University Medical Centre healthy volunteers biobank. Written informed consent of the volunteers was obtained.

Blood samples were collected in Vacuette^®^ sodium citrate 3.8% blood collecting tubes (Greiner Bio-one, GmbH, Kremsmünster, Austria). Within an hour after venipuncture, the blood samples were centrifuged to separate the plasma from the cells. Plasma samples were aliquoted and stored at −80 °C pending analysis.

### 4.2. Immunoassay AαVal541

To study the course of the AαVal541 levels, a specific Europium-based immunoassay was developed to measure the PR3-generated fibrinopeptide neo-epitope AαVal541. The Val541 cleavage site on the alpha chain of fibrinogen by PR3 was previously described by Newby et al. [11].

Antiserum against the carboxyl amino acid side of AαVal541 was generated in New Zealand white rabbits (Rockland Immunochemicals, Limerick, PA, USA). The synthetic peptide Cys-Orn-Met-Leu-Gly-Glu-Phe-Val (Rockland Immunochemicals) was conjugated to maleimide-modified bovine thyroglobulin for the immunization in rabbits by Rockland Immunochemicals. The unconjugated peptide was used as an antigen in the immunoassay for generating a standard curve in the range of 5000–1.3 nM.

For this assay, high binding white 96-well microplates (Thermo Fisher Scientific, Waltham, MA, USA) were coated with PR3-cleaved fibrinogen. Cleaved fibrinogen was generated by incubating human fibrinogen (Haemocomplettan P; CSL Behring BV, Marburg, Germany) with PR3 (Athens Research and Technology, Athens, GA, USA). Human fibrinogen diluted in 150 mM NaCl–50 mM Tris pH 7.5 buffer was incubated with PR3 at 37 °C in a molar ratio of 1:400 (PR3: fibrinogen). The cleavage reaction was stopped with excess AAT (Respreeza^®^, CSL Behring BV, Marburg, Germany) in molar ratio 1:200 (PR3: AAT). Appropriately diluted PR3-cleaved fibrinogen (100 µL) in 15 mM sodium carbonate–35 mM sodium bicarbonate buffer was added to each well and incubated overnight at 4 °C. In a 96-well non-binding plate (Greiner Bio-One GmbH, Kremsmünster, Austria), 100 µL of the unconjugated synthesized peptide (Cys-Orn-Met-Leu-Gly-Glu-Phe-Val; Rockland Immunochemicals) or 100 µL diluted plasma samples (diluted in blocking/dilution buffer: 1% (*w*/*v*) bovine serum albumin (BSA) Tris-buffered-saline, 0.02% (*v*/*v*) Tween-20 (TBS)) was added together with 100 µL of the rabbit antiserum diluted in 1% BSA-TBS and incubated overnight at 4 °C. The next day, the antigen-coated plate was washed with DELFIA wash buffer (Perkin Elmer, Waltham, MA, USA) followed by incubation with blocking buffer 1% BSA-TBS for one hour at 37 °C to prevent non-specific binding. After incubation, the plate was washed again with DELFIA wash buffer. The pre-incubated samples were added to the washed plate and incubated for two hours at 37 °C. Next, the plate was washed with DELFIA wash buffer followed by the addition of DELFIA Europium-conjugated goat-anti-rabbit IgG (Perkin Elmer) diluted in 1% BSA-TBS and incubation for one hour at RT. A final wash with DELFIA wash buffer was performed, and DELFIA Enhancement solution (Perkin Elmer) was added for the development of the fluorescence signal. The plate was read at excitation 370 nm and emission 615 nm. The signal was measured with a 400-microsecond delay and counted for 400 micro-seconds using a Spectramax i3x (Molecular Devices, San Jose, CA, USA). By applying a 4-parametric mathematical fit model using GraphPad Prism 8 [24], a standard curve was generated to interpolate the samples. All the different dilutions of a plasma sample were used to calculate the level of AαVal541 if the measured value of the diluted sample felt in the 20–80% binding range of the standard curve.

### 4.3. Western Blot Method

For the Western blot analysis, 0.25 µg of different preparations of cleaved fibrinogen were analyzed. The samples were first diluted in 50 mM Tris–150 mM NaCl pH7.5 buffer. The final dilution was made in reducing SDS sample buffer (Thermo Fisher Scientific, Waltham, MA, USA) and heated for 5 min at 100 °C. Next the samples were added to 4–15% mini-PROTEAN TGX precast 10-well protein gel (Bio-Rad, Hercules, CA, USA) together with Page ruler Plus prestained protein ladder (Thermo Fisher Scientific). Subsequently, the proteins were blotted on a polyvinylidene fluoride (PVDF) membrane (Trans-blot Turbo Bio-Rad), and non-specific binding sites were blocked in phosphate-buffered saline containing 0.1% (*v*/*v*) Tween- 20 (PBST) and 5% (*w*/*v*) BSA. After blocking, the AαVal541 antibody in a 1:5000 dilution made in PBST 5% BSA was incubated with the PVDF membrane. Next, goat-anti-rabbit-HRP conjugated antibody in a 1:10,000 dilution made in PBST 5% BSA was added. Signal was developed with ECL (Thermo Fisher Scientific), and visualized by using the ChemiDoc™ Touch imager (Bio-Rad); Image Lab™ software [25] was used to analyze the image.

### 4.4. AAT Levels

AAT plasma levels in gram/liters (g/L) were measured at the Clinical Chemistry Laboratory of the Leiden University Medical Center by the immunoturbidimetric method, at wavelength 340 nm, on the Cobas 8000 (Roche Diagnostics, Basel, Switzerland).

### 4.5. Pharmacokinetic Model Development and Model Validation

Nonlinear mixed effect modeling was used to estimate AAT pharmacokinetic parameters from plasma concentration-time data. NONMEM (v7.4.4, Icon Development Solutions, Ellicott City, MD) was used for modeling AAT pharmacokinetics, using PsN toolkit (v5.0.0) [26,27,28] and Pirana version 2.9.8 [29] as modeling environment. R statistics (v. 3.4.4) was used for exploratory graphical analysis and for evaluation of the goodness-of-fit plots (GOF) and prediction-corrected visual predictive check (pcVPC) [30]. First-order conditional estimation method with interaction (FOCE-I) was used throughout the analysis. Model selection was based on statistical significance, goodness-of-fit and stability. Throughout the model building process, an alternative model was selected to replace a previous model if a difference in the objective functions (−2 log likelihood) was >6.63 (*p* < 0.01, with 1 degree of freedom, assuming (Chi-squared) X^2^ distribution). The final model was evaluated and validated by means of a pcVPC based on 500 Monte Carlo simulations. In addition, the precision of the parameter estimates was further assessed by means of a nonparametric bootstrap with resampling the dataset (*n* = 1000 times). A graphical display of the structure of the population pharmacokinetic model is presented in Figure S7.

### 4.6. Statistical Analysis

Statistical analyses were performed using IBM SPSS version 25 [31] and GraphPad Prism 8 [24]. All graphs, except for the pharmacokinetic graphs, were generated using GraphPad Prism 8 [24]. Data of our study subjects were not normally distributed, and therefore continuous data are expressed in median with interquartile range (IQR) and categorical data as numbers and percentages. To test for differences between groups the Mann–Whitney U test was performed. Correlation analysis was done by the Spearman correlation coefficient. Differences with a *p*-value < 0.05 were considered statistically significant.

## 5. Conclusions

In conclusion, our results show that fibrinopeptide AαVal541 may serve as a biochemical footprint for assessing the efficacy of in vivo inhibition of proteinase 3 activity in AATD patients receiving intravenous AAT augmentation therapy. This direct reflection of the effect of AAT augmentation therapy opens opportunities for personalized and tailored AAT dosing, which might support a potential beneficial effect on pulmonary function over the long term.

## Figures and Tables

**Table 1:** Characteristics of study population. The data are presented as median with IQR and in percentages. None of the AATD patients had a history of an acute exacerbation 6 weeks prior to pulmonary function testing. The group of healthy controls were comparable qua age and gender to the AATD patients Abbreviations: IQR = interquartile range; FEV_1_ = forced expiratory volume in one second; FVC = forced vital capacity; KCO = transfer coefficient of the lung for carbon monoxide.

	AATD	Healthy Controls
Subjects	10	20
Age (median, IQR)	57.5 (52.5–63.3)	54 (47–60.5)
Gender (%)		
Female	7 (70%)	13 (65%)
Male	3 (30%)	7 (35%)
Ex-smokers (%)	6 (60%)	*Unknown*
Current smokers (%)	0 (0%)	0 (0%)
Genotype (%)		
Z/Q0_Bellingham_	2 (20%)	
Z/Q0_Amersfoort_	1 (10%)	
Z/M_Procida_	2 (20%)	
Z/M_Heerlen_	2 (20%)	
Q0_Amersfoort_/M_Heerlen_	2 (20%)	
M_Heerlen_/M_Heerlen_	1 (10%)	
Post bronchodilator FEV_1_ in L	1.6 (1.2–2.4)	
Post bronchodilator FEV_1_ in %predicted	54.5 (41.3–78.8)	
FEV_1_/FVC-ratio %	49.8 (35.2–69.3)	
KCO %predicted	66 (36.8–71.3)	
6 min walking test in meters	484 (392.3–525.5)	

**Table 2:** Estimated pharmacokinetic parameters of the two-compartment population pharmacokinetic model of a single dose of 240 mg/kg AAT.

Parameter	Value
CL (L/day)	0.605
V_central_ (L)	3.14
Q (L/day)	2.11
V_peripheral_ (L)	3.09
Inter-individual variability CL (CV%)	23.4
Inter-individual variability V_central_ (CV%)	42.4
Inter-individual variability Q (CV%)	118.4

CL = clearance of AAT; V_central_ = volume of distribution central; Q = intercompartmental clearance; V_peripheral_ = volume of distribution peripheral; CV% = coefficient of variation.

**Table 3:** Pharmacokinetic parameters of each individual AAT patient after applying the pharmacokinetic modelling.

Patient	AUC_0-inf_	C_av_ (g/L)	C_max_ (g/L)	Max Decrease AαVal541 (% of Baseline)	*x*-Time Infusion	Age (years)	Weight (kg)
1	38.0	1.6	5.0	96.2	1st	57	72.9
2	28.0	1.2	5.2	87.2	1st	58	52.1
3	20.5	0.9	6.4	95.9	1st	53	81.9
4	27.8	1.2	5.3	49.9	5th	55	75.6
5	18.3	0.8	4.4	87.1	1st	51	65.5
6	29.3	1.2	6.8	92.3	1st	50	79.8
7	26.9	1.1	5.0	75.4	4th	63	58.0
8	42.1	1.8	9.2	57.3	2nd	65	92.0
9	29.6	1.2	6.1	74.3	4th	64	69.2
10	23.6	1.0	5.0	83.2	4th	59	59.0

AUC_0-inf_ = area under the AAT plasma concentration-time curve from dose administration to infinity; C_av_ = Average concentration AAT in g/L; C_max_ = Peak plasma concentration of AAT in g/L; Max decrease AαVal541 % of baseline = the maximum decrease of the AαVal541 after infusion of AAT in percentage of the AαVal541 level before infusion.

## Data Availability

The data presented in this study are available on request.

## Supplementary Materials

The following are available online at https://www.mdpi.com/article/10.3390/ijms22158031/s1.
