# Supplementary material for: The Course of AαVal541 as a Proteinase 3 Specific Neo-Epitope after Alpha-1-Antitrypsin Augmentation in Severe Deficient Patients
Source: Int J Mol Sci. 2021 Jul 27;22(15):8031. doi: 10.3390/ijms22158031 (PMC8347723; doi:10.3390/ijms22158031)
Supplement: Supplementary file 1 [file ijms-22-08031-s001.zip › ijms-1285151-supplementary.pdf]

Supplementary material

The course of AαVal541 as a proteinase 3 specific neo-epitope after alpha-1-antitrypsin augmentation in severe deficient patients

Iris G. M. Schouten[1],*, Richard A. Mumford[1], Dirk Jan A. R. Moes[2], Pieter S. Hiemstra[1], Jan Stolk[1]

1 Department of Pulmonology, Leiden University Medical Center, Leiden, The Netherlands;

2 Department of Clinical Pharmacy and Toxicology, Leiden University Medical Center, Leiden, The Netherlands

* Correspondence: Iris G. M. Schouten; i.g.m.schouten@lumc.nl

Index

1. Development of AαVal541 immunoassay
   1.1. Antisera development
   1.2. Specificity of antibody
2. Individual AαVal541 graphs
3. Individual pharmacokinetic model based graphs
4. Correlation of pharmacokinetic parameters
5. Graphical representation pharmacokinetic model

1. Development of AαVal541 immunoassay

1.1 Antisera selection

The antisera of two rabbits were tested at different dilutions in the immunoassay. The results obtained using both antisera were comparable and the sera from the 972 rabbit was selected for further use. See Figure S1.

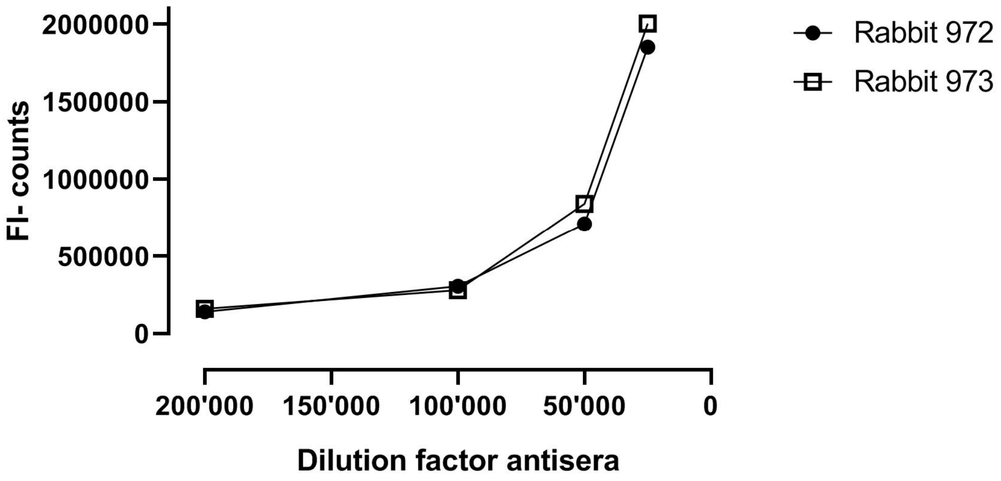

Figure S1. **Comparison of AαVal541 antisera of two different immunised rabbits.** The antisera against AαVal541 generated in two rabbits were added in different dilutions to the plates coated with PR3- cleaved fibrinogen. Although there was no clear distinction in total fluorescence counts between the antisera of the two rabbits, the antiserum of rabbit 972 was for use in the immunoassay based on the very small difference at the higher dilutions.
Abbreviations: Fl= fluorescence counts

1.2 Specificity of AαVal541 antibody

The specificity of the antibody for the AαVal541 epitope which is generated by PR3 was tested using PR3-cleaved fibrinogen, NE-cleaved fibrinogen and uncleaved fibrinogen by competitive immunoassay and western blot analysis. The NE-cleaved fibrinogen was prepared as follows: NE (Athens Research and Technology, Athens, Georgia, USA) was diluted in 50mM Na acetaat-150mM NaCl, pH 5.5 buffer and incubated with human fibrinogen (Haemocomplettan P; CSL Behring BV) at room temperature in a molar ratio of 1:200. The reaction was stopped by adding the synthetic low molecular weight NE- and PR3 inhibitor DMP-777 (Merck & Co, Inc Rahway, NJ, The United States)

diluted in DMSO in molar ratio 1:925 (PR3: DMP-777).

The total fluorescence counts after adding the PR3-cleaved fibrinogen, NE-cleaved fibrinogen and uncleaved fibrinogen in the immunoassay were compared (Figure S2). The concentration of fibrinogen used to coat the plate in the assay is around 2.3nM. There appeared to be no binding of the AαVal541 antibody to uncleaved fibrinogen at this concentration, but there was some binding of the AαVal541 antibody to NE-cleaved fibrinogen.

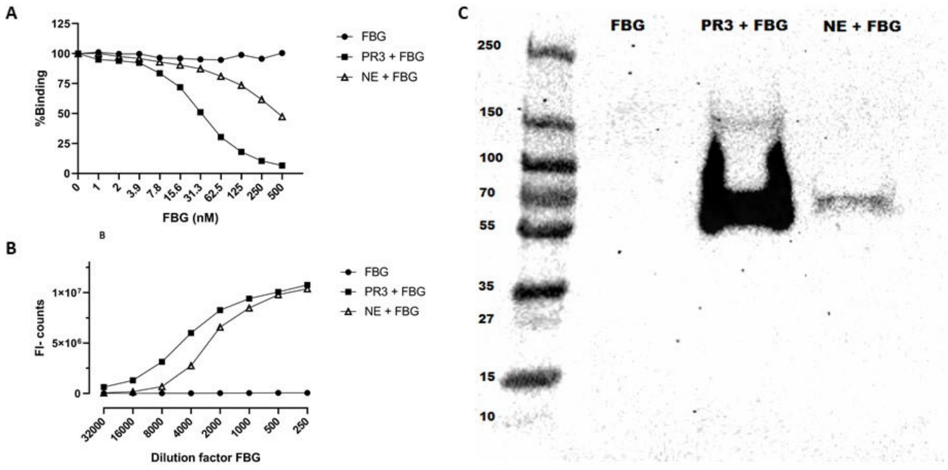

Figure S2. AαVal541 antibody specificity for the AαVal541 fibrinogen neo-epitope. (**A)** Uncleaved fibrinogen, NE- and PR3-cleaved fibrinogen were incubated overnight with the AαVal541 antibody, and the next day added to a plate coated with PR3-cleaved fibrinogen. Total fluorescence counts were measured after adding DELFIA europium-conjugated goat-anti-rabbit IgG and DELFIA enhancement solution. **(B)** Uncleaved fibrinogen, NE-and PR3-cleaved fibrinogen were coated to the plate in a dilution of 1:20’000 and next incubated with the AαVal541 antibody. Binding was detected as in (A). **(C)** Binding of the AαVal541 antibody to uncleaved fibrinogen, NE- and PR3-cleaved fibrinogen was also determined by western blot analysis. **Abbreviations:** FBG= fibrinogen, NE= neutrophil elastase, PR3= Proteinase 3, Fl= fluorescence

The specificity of the AαVal541 antibody was also tested by using various modifications of the AαVal541 peptide, and using the NE-specific epitope AαVal360 (H-Cys-Nle- Thr-Ser-Glu-Ser-Ser-Val-OH). (*US patent No. 6124107*)*.* The valine at the N-terminal of the AαVal541 peptide was crucial for detection of the AαVal541 antibody. The AαVal360 epitope was not recognized by the AαVal541 antibody (Figure S3).

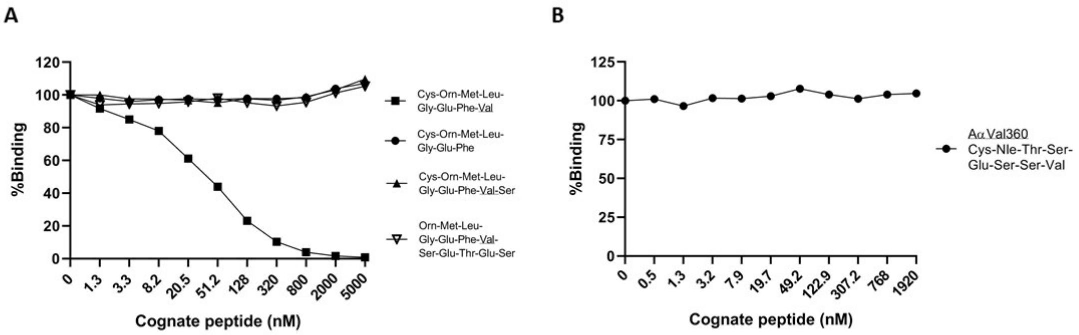

Figure S3. AαVal541 antibody specificity for AαVal541 epitope. (A) Different modifications of the AαVal541 cognate peptide were evaluated in the immunoassay by incubating them with the AαVal541 antibody to a plate coated with PR3-cleaved fibrinogen. **(B)** Same as A but with NE-specific fibrinogen epitope AαVal360 (H-Cys-Nle- Thr-Ser-Glu-Ser-Ser-Val-OH) (*US patent No. 6124107*)

2. Individual AαVal541 graphs

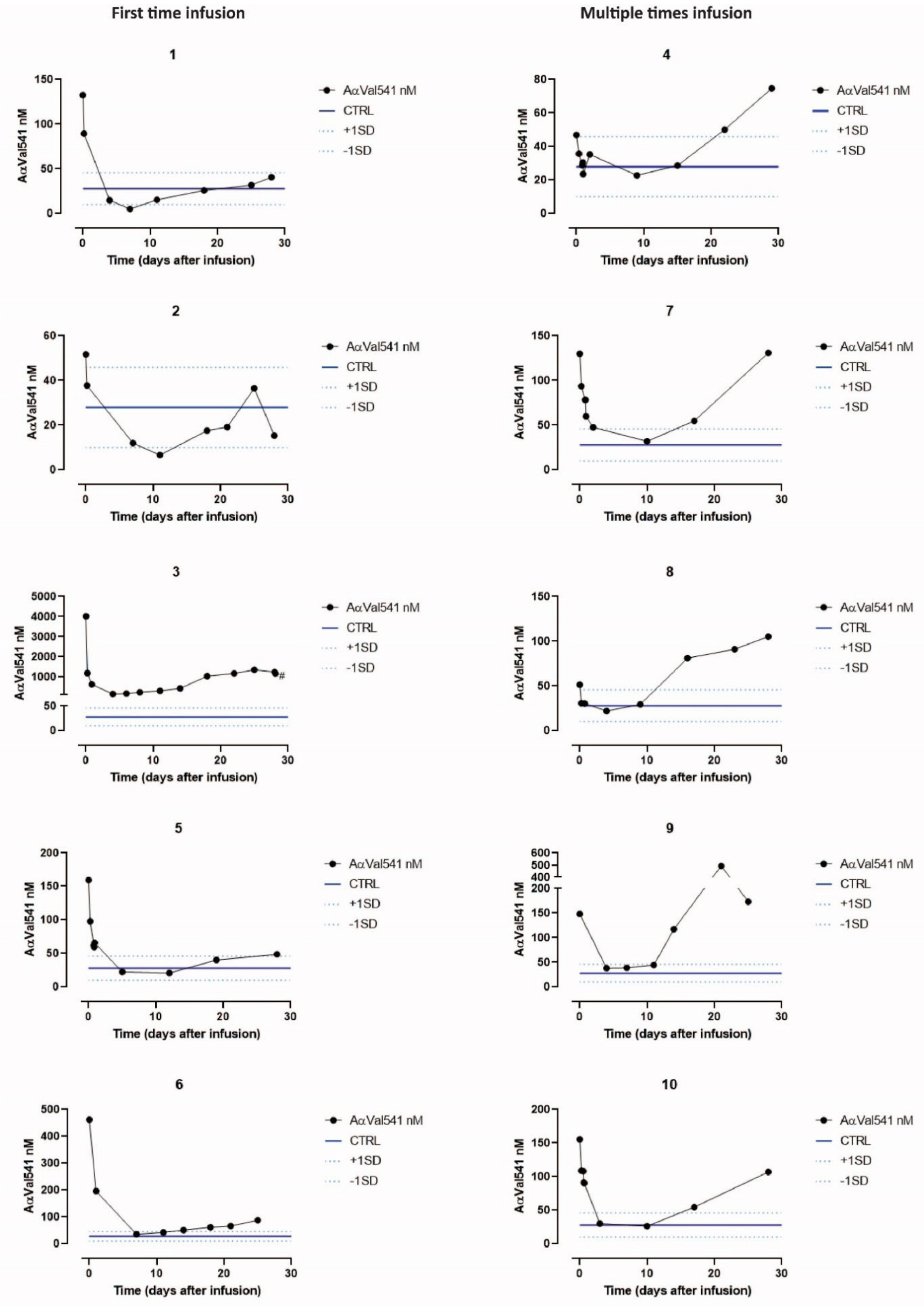

Figure S4. The course of AαVal541 after single dose of AAT 240mg/kg plotted for each individual together with the range of AαVal541 found in healthy individuals # At this point, another infusion of AAT of 240mg/kg was administered.

3. Individual pharmacokinetic models graphs

The individual graphs of the pharmacokinetic modeling are shown below in Figure S5.

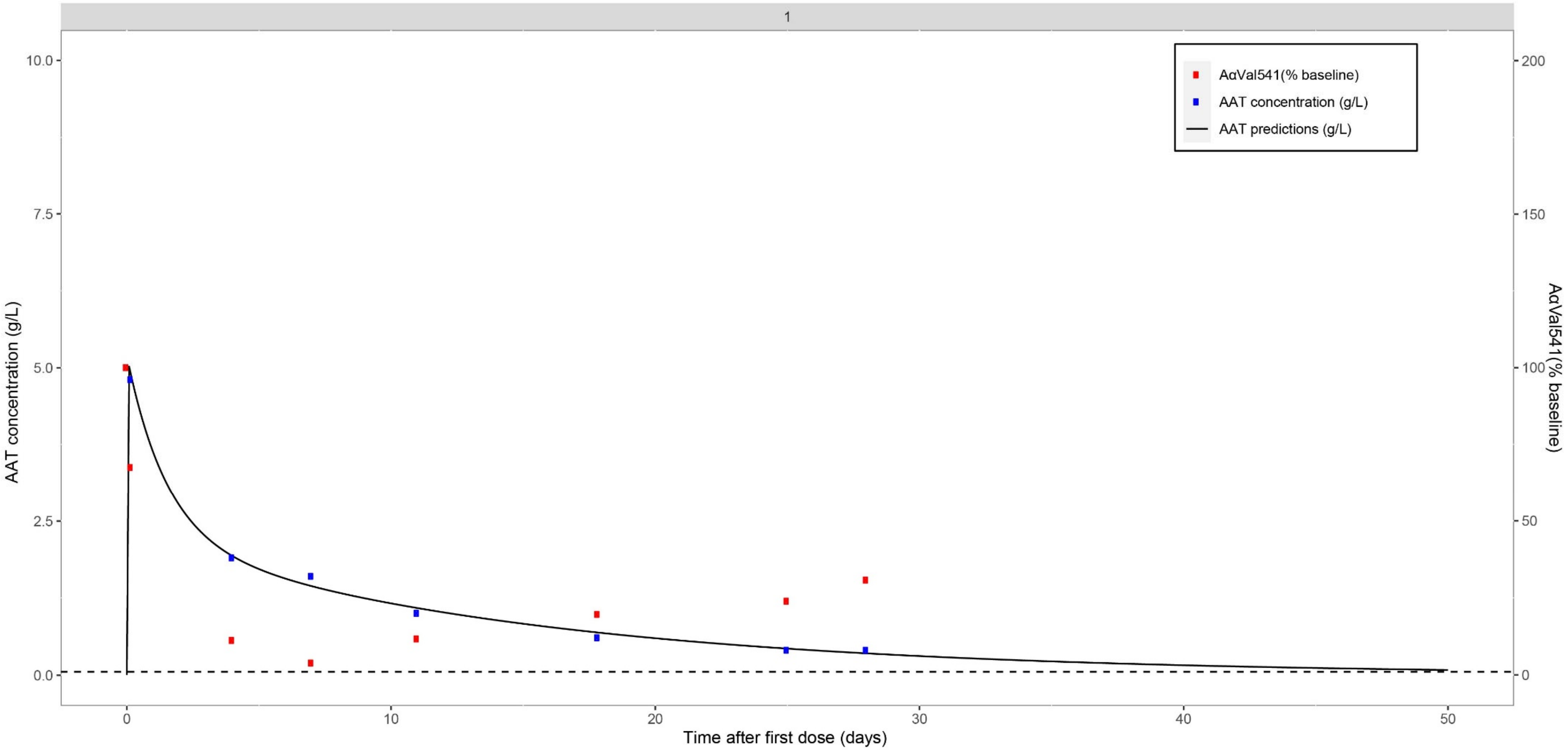

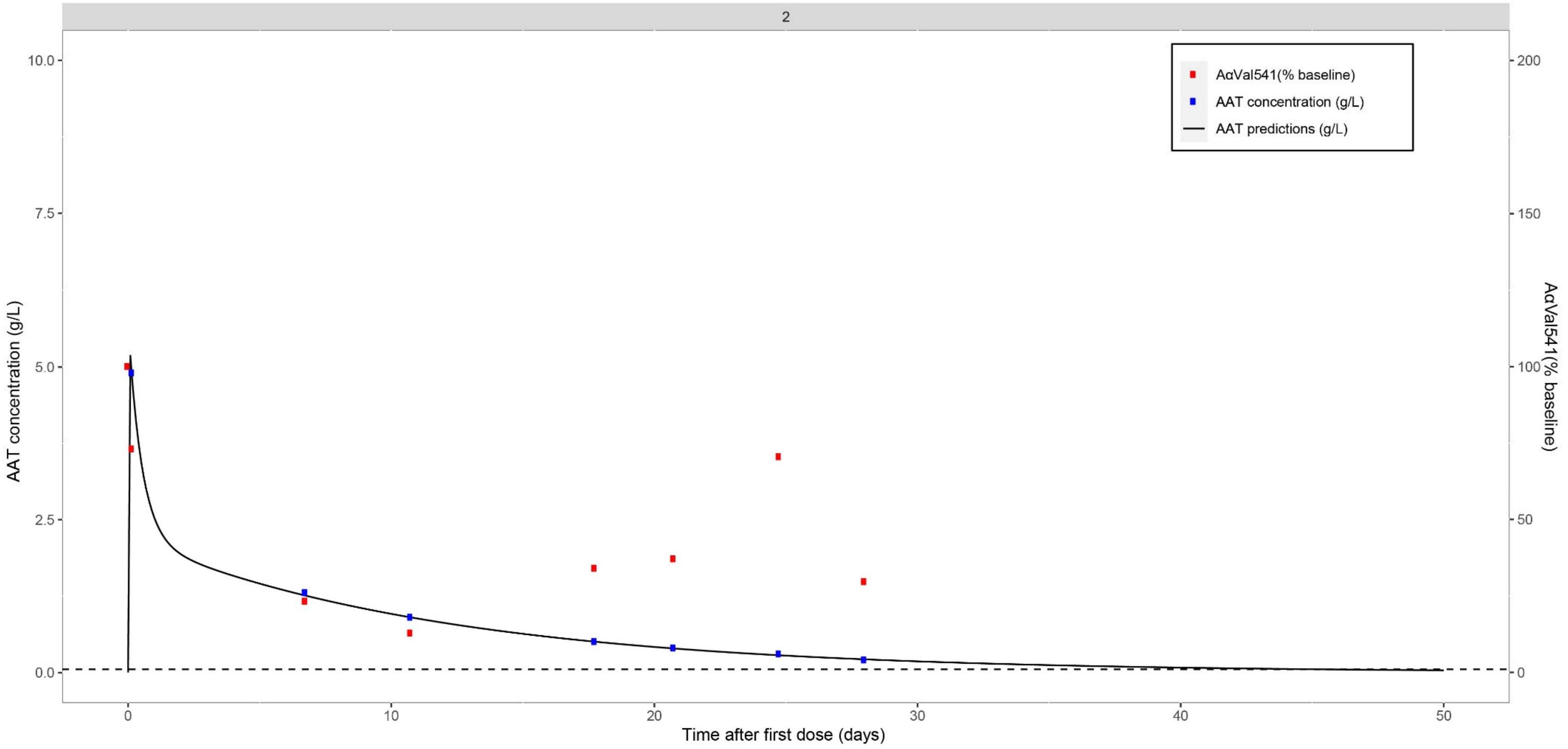

2
AαVal541(% baseline)
AAT concentration (g/L)
AAT predictions (g/L)
AAT concentration (g/L)
AαVal541(% baseline)
Time after first dose (days)
10.0
7.5
5.0
2.5
0.0
200
150
100
50
0
0
10
20
30
40
50

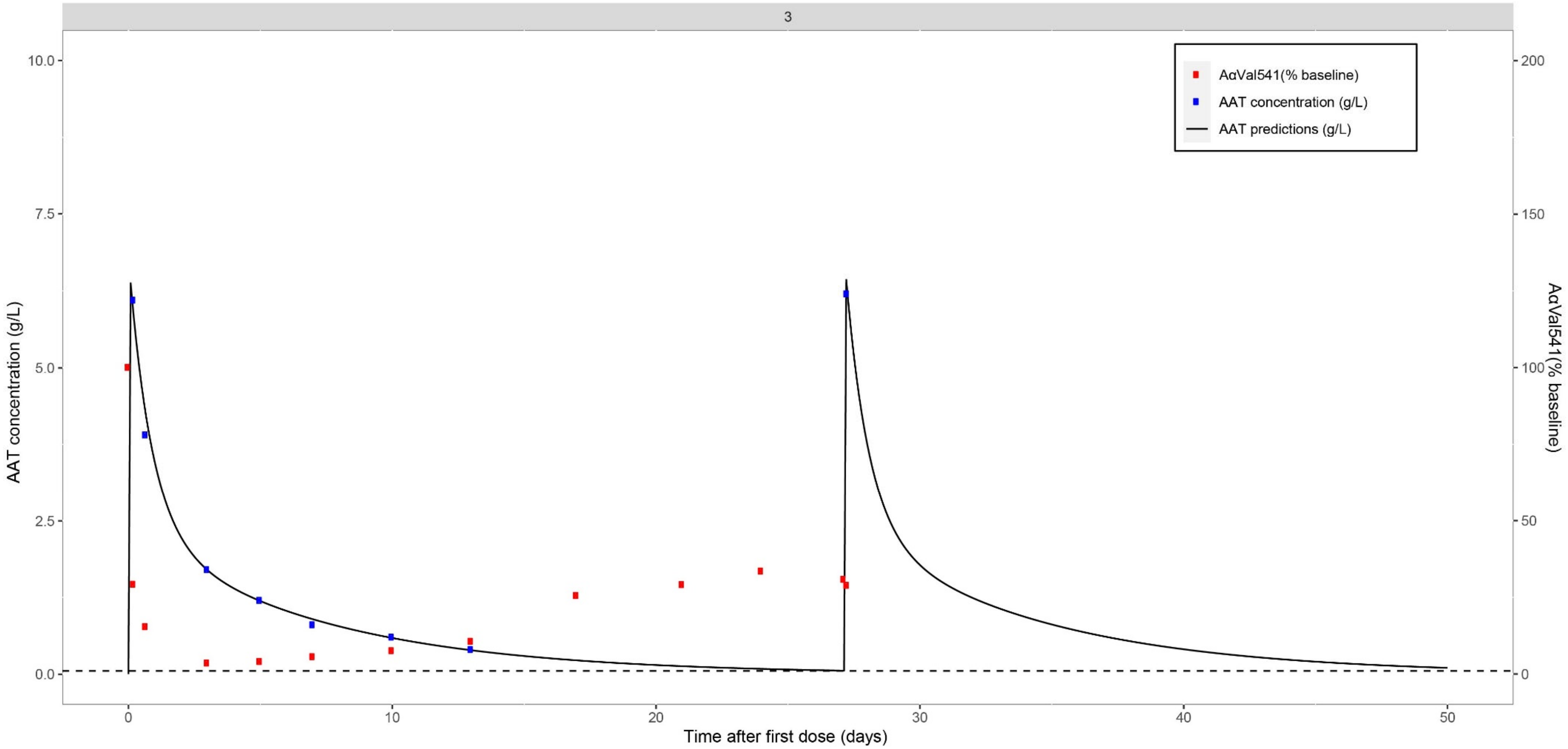

3
AαVal541(% baseline)
AAT concentration (g/L)
AAT predictions (g/L)
AAT concentration (g/L)
AαVal541(% baseline)
Time after first dose (days)
10.0
7.5
5.0
2.5
0.0
200
150
100
50
0
0
10
20
30
40
50

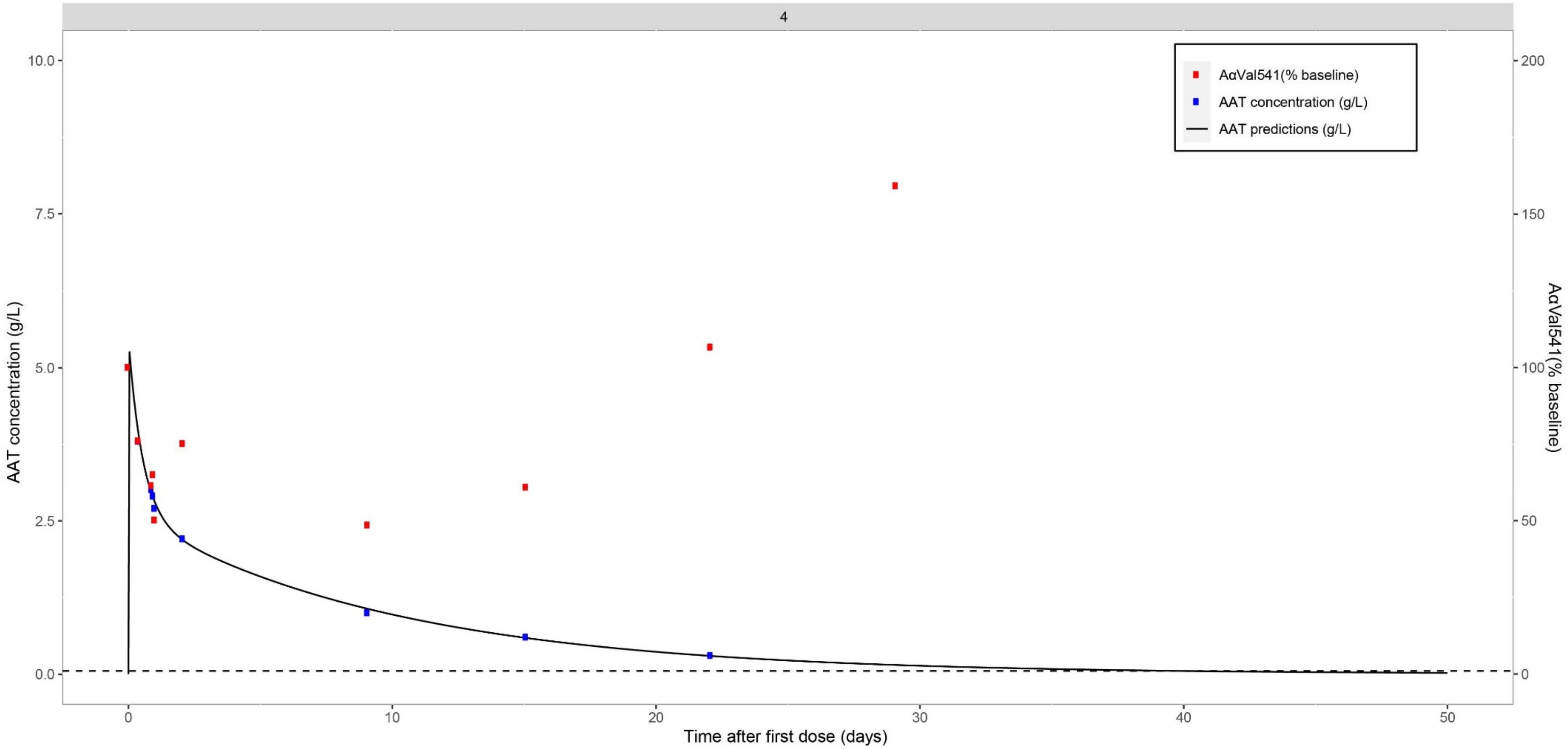
4
AαVal541(% baseline)
AAT concentration (g/L)
AAT predictions (g/L)
AAT concentration (g/L)
AαVal541(% baseline)
Time after first dose (days)
10.0
7.5
5.0
2.5
0.0
200
150
100
50
0
0
10
20
30
40
50

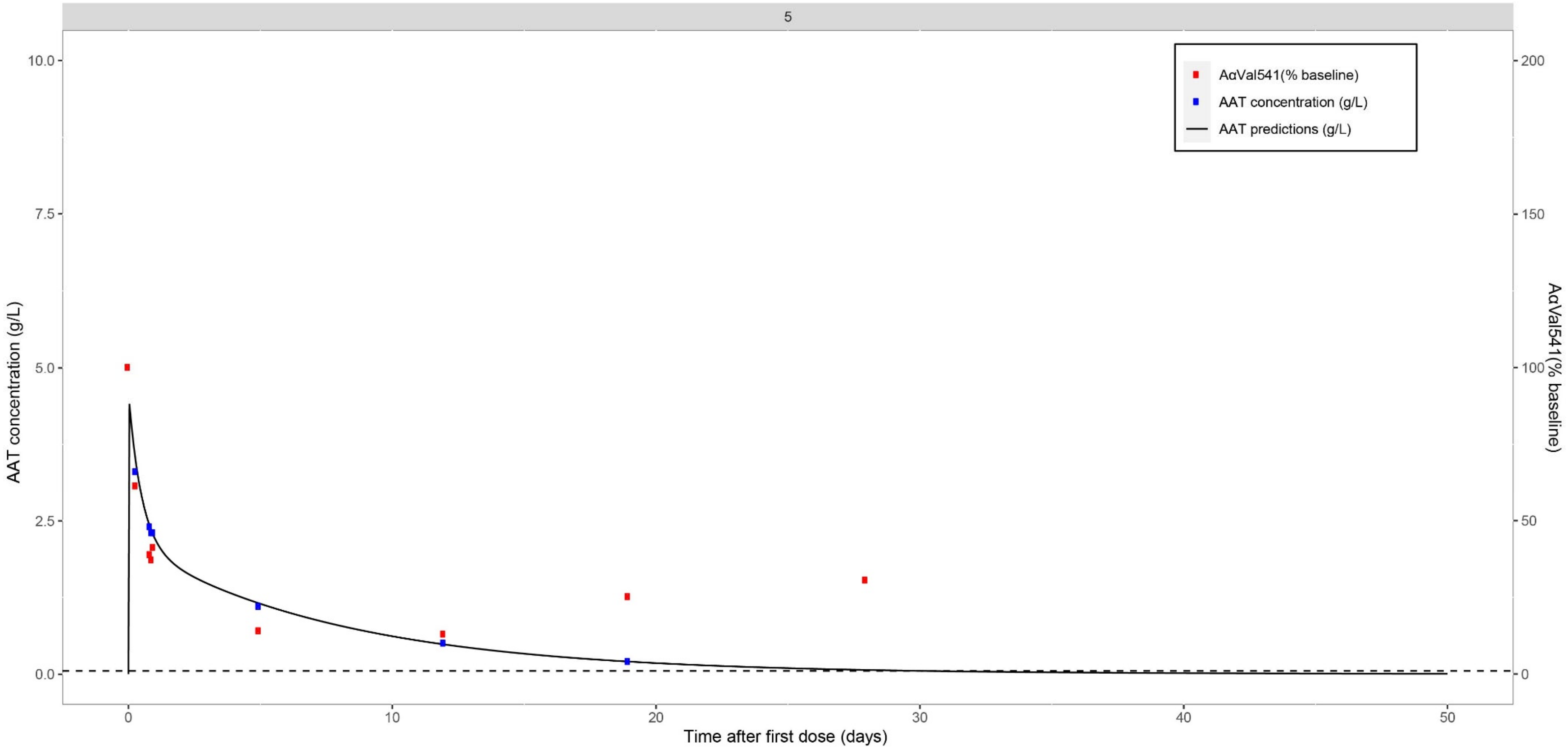

5
AαVal541(% baseline)
AAT concentration (g/L)
AAT predictions (g/L)
10.0
7.5
5.0
2.5
0.0
AAT concentration (g/L)
200
150
100
50
0
AαVal541(% baseline)
0
10
20
30
40
50
Time after first dose (days)

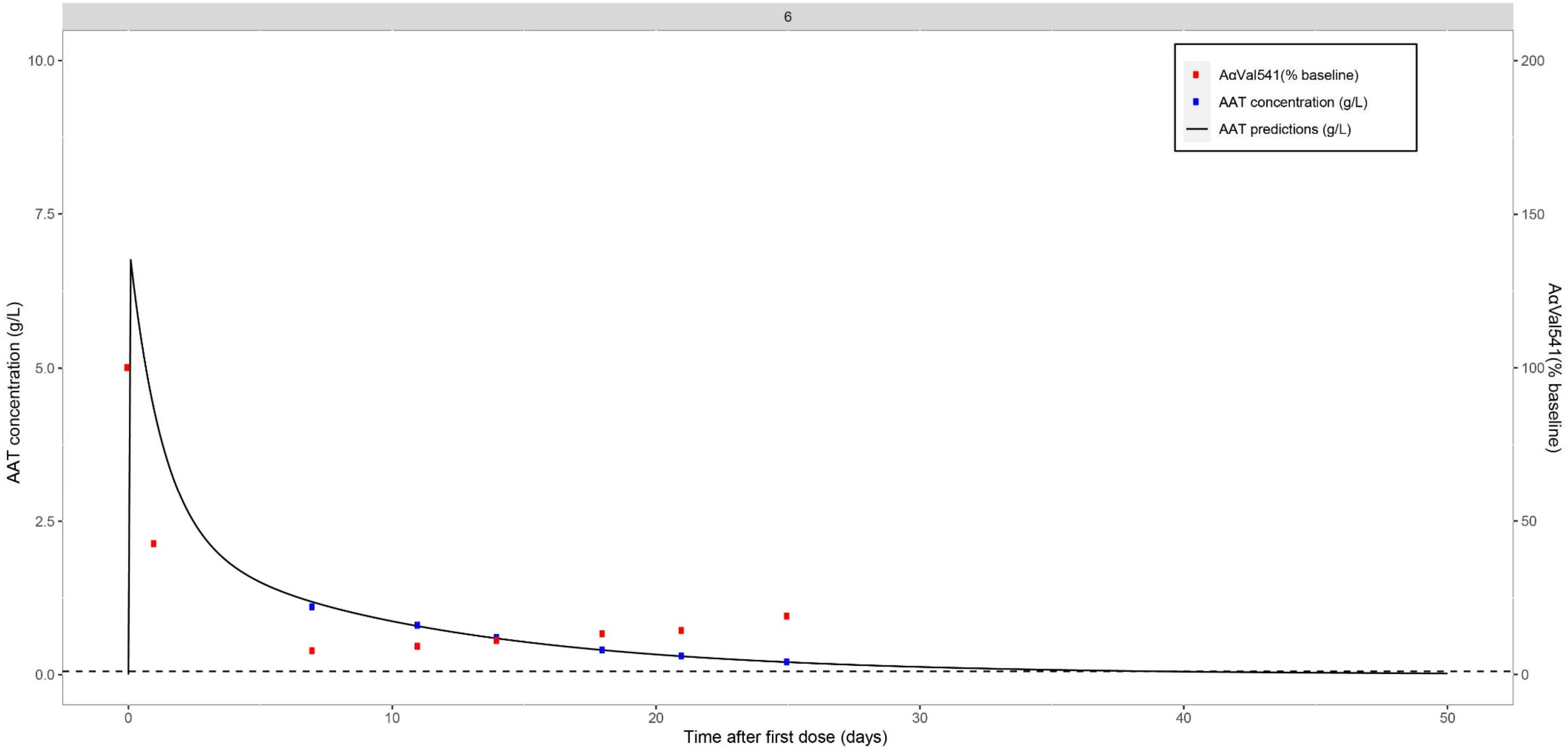

6
10.0
7.5
5.0
2.5
0.0
AAT concentration (g/L)
200
150
100
50
0
AαVal541(% baseline)
AαVal541(% baseline)
AAT concentration (g/L)
AAT predictions (g/L)
0
10
20
30
40
50
Time after first dose (days)

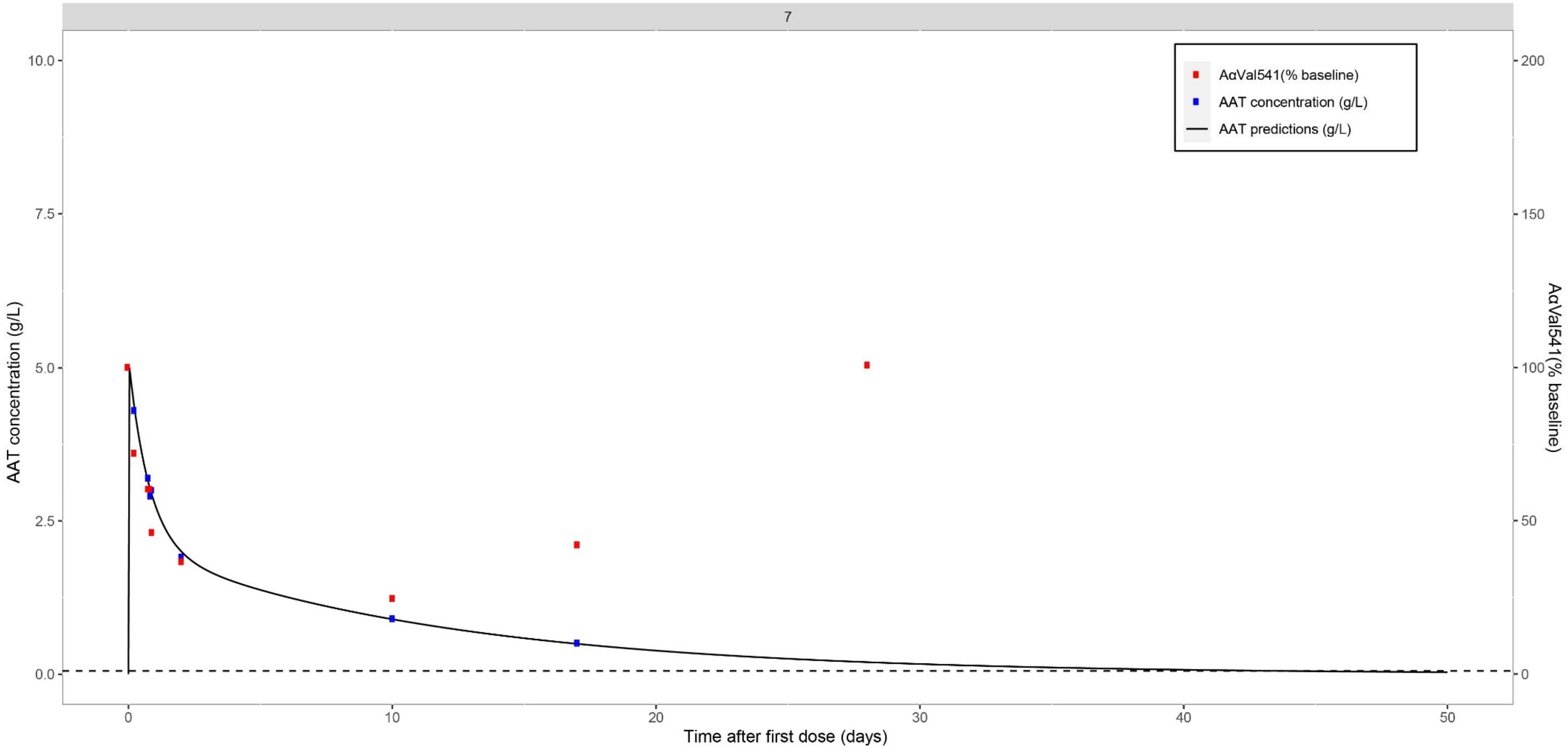

7
AαVal541(% baseline)
AAT concentration (g/L)
AAT predictions (g/L)
10.0
7.5
5.0
2.5
0.0
AAT concentration (g/L)
200
150
100
50
0
AαVal541(% baseline)
0
10
20
30
40
50
Time after first dose (days)

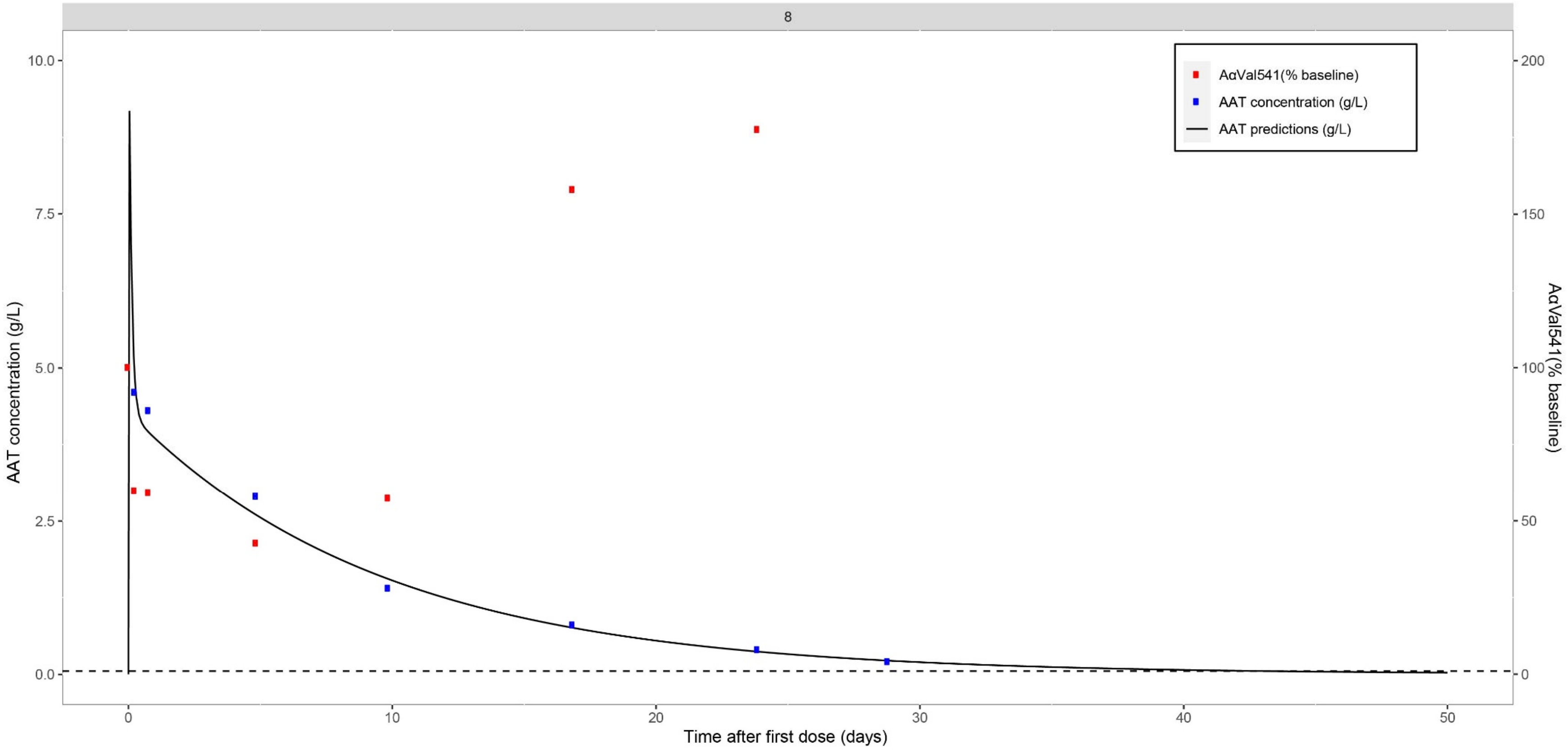
8
AαVal541(% baseline)
AAT concentration (g/L)
AAT predictions (g/L)
AAT concentration (g/L)
AαVal541(% baseline)
Time after first dose (days)
10.0
7.5
5.0
2.5
0.0
200
150
100
50
0
0
10
20
30
40
50

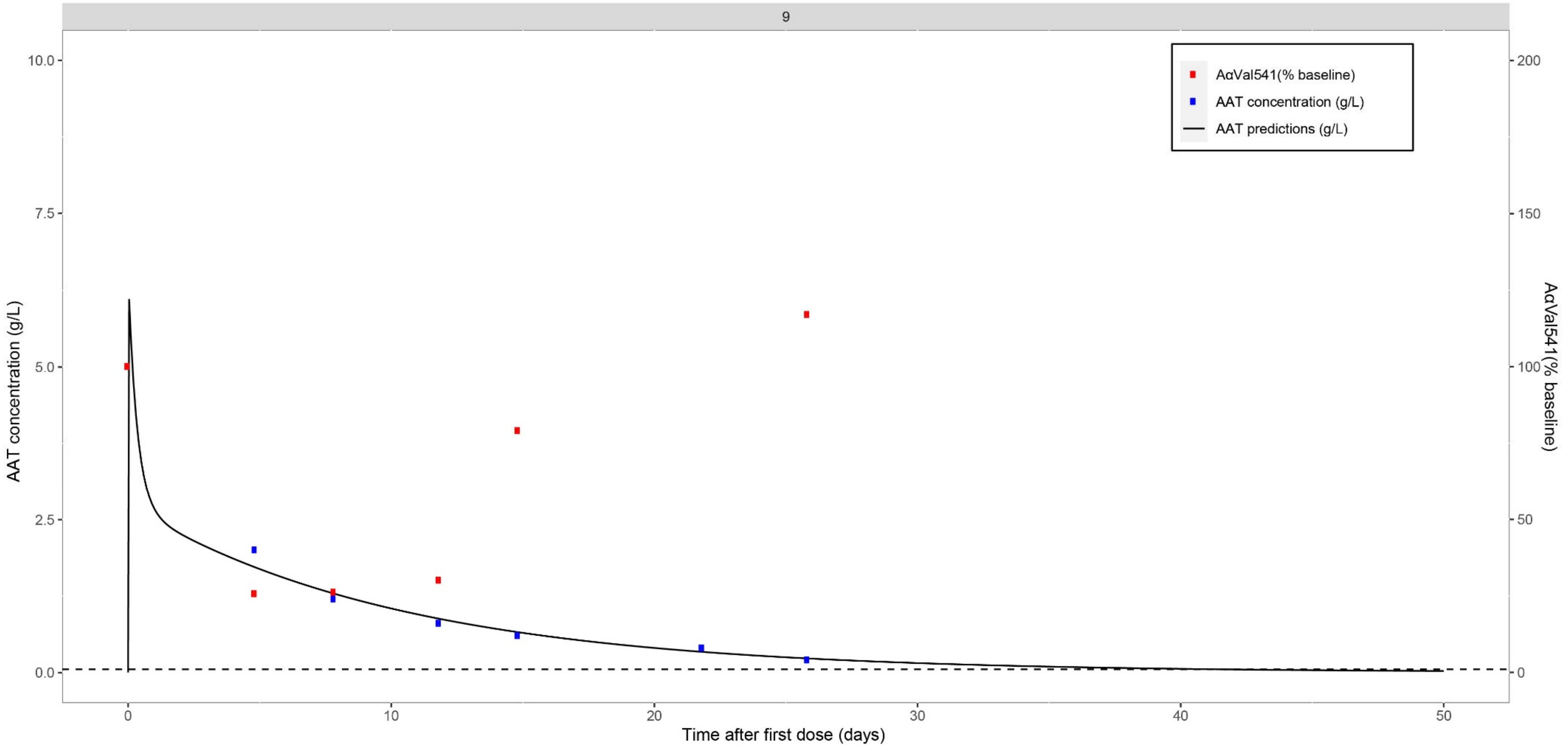
9
AαVal541(% baseline)
AAT concentration (g/L)
AAT predictions (g/L)
AAT concentration (g/L)
AαVal541(% baseline)
Time after first dose (days)
10.0
7.5
5.0
2.5
0.0
200
150
100
50
0
0
10
20
30
40
50

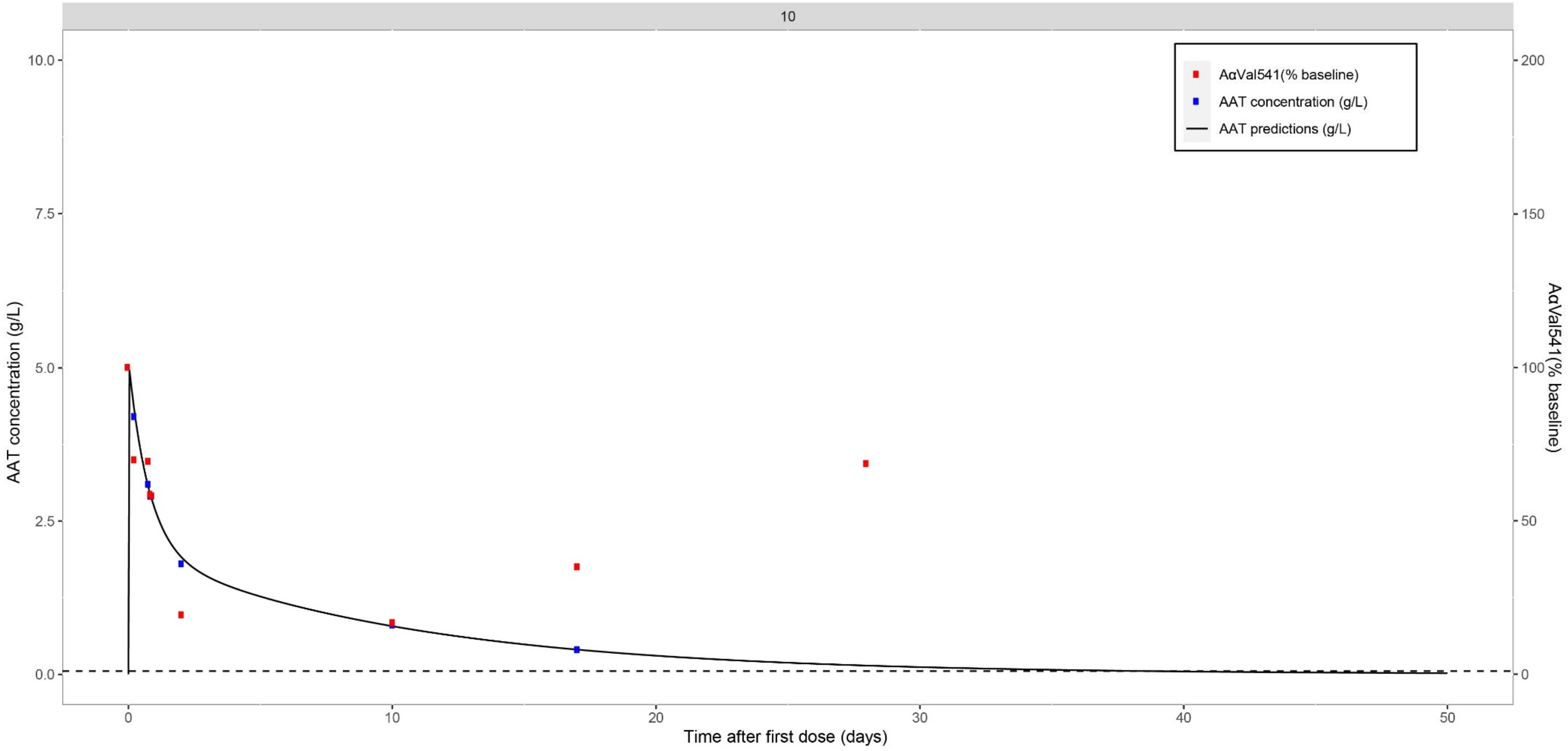

Figure S5. Individual graphs. The Left y-as plots the AAT levels with both the predicted by applying two- compartment pharmacokinetic model (black line) and the measured (blue dots) AAT levels at different time points over a month, the right y-as plots the AαVal541 levels (red dots) expressed as percentage of baseline (AαVal541 before the infusion) measured on the different time points.

4. Pharmacokinetic modeling graphs

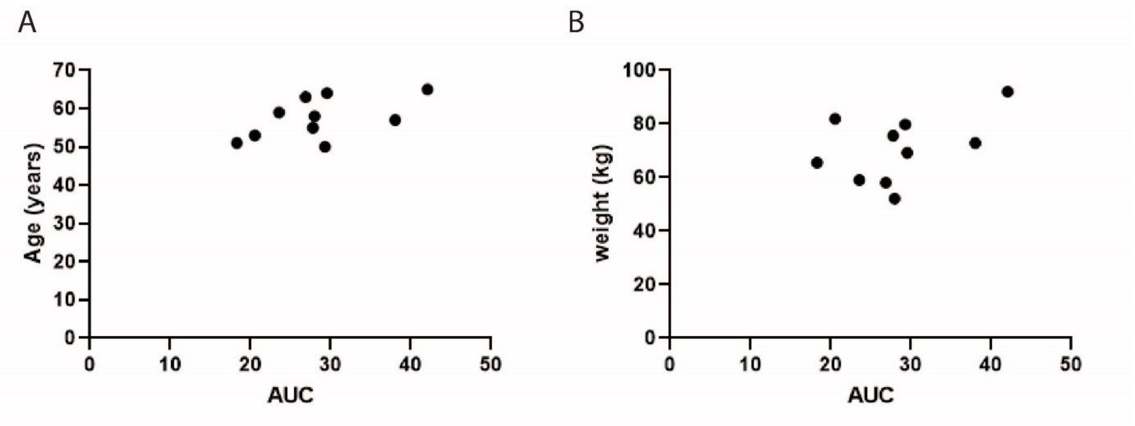

Figure S6. Correlation of $AUC_{0\text{-}inf}$ with subjects age and weight. In (**A**) the age of each patient is plotted against the AUC. There was no correlation between age and $AUC_{0\text{-}inf}$. (Spearman coefficient= 0.4667, p= 0,1786). In (**B)** the weight of each individual is plotted against $AUC_{0\text{-}inf}$. There was no correlation between weight and $AUC_{0\text{-}inf}$. (Spearman coefficient= 0.333, p=0.347).
Abbreviations: $AUC_{0\text{-}inf}$= area under the AAT plasma concentration- time curve from dose administration to infinity.

5. Graphical representation population pharmacokinetic model

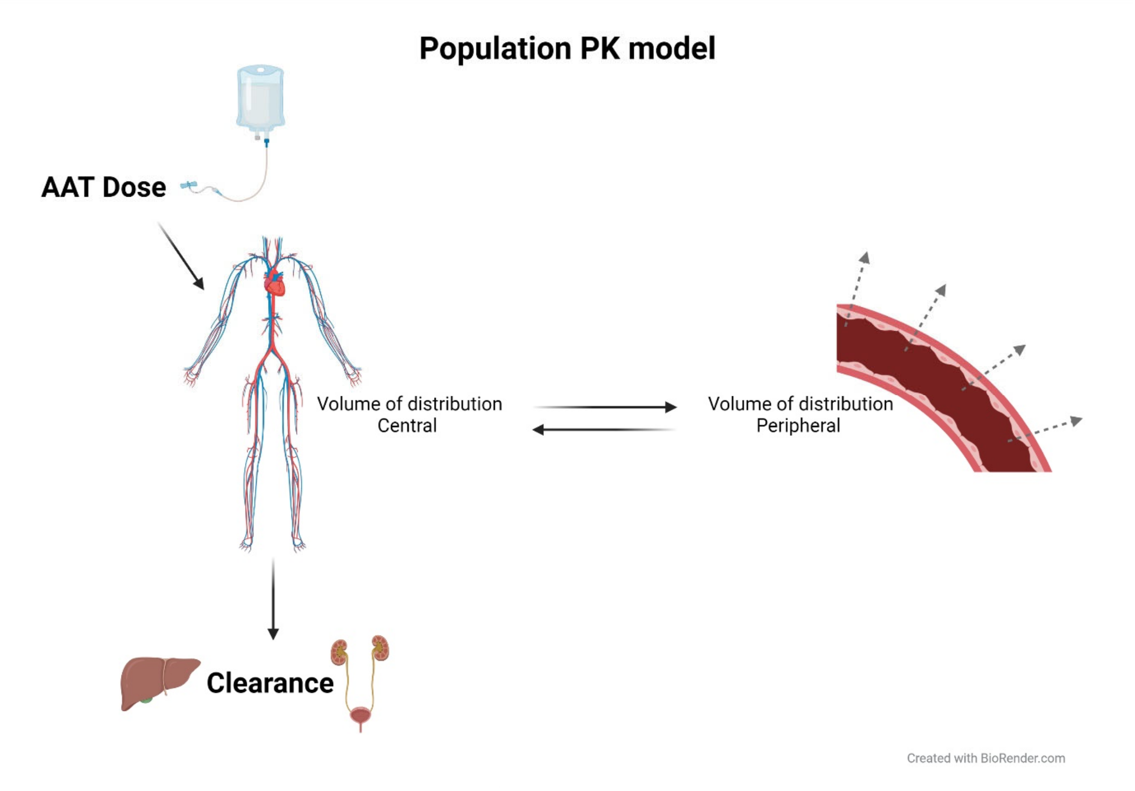

Figure S7. The graphical display of the structure and interactions of the population pharmacokinetic model.
